# Robot - assisted laparoscopic retroperitoneal lymph node dissection in testicular tumor

**DOI:** 10.1590/S1677-5538.IBJU.2015.0436

**Published:** 2017

**Authors:** Fabio C. M. Torricelli, Denis Jardim, Giuliano B. Guglielmetti, Vipul Patel, Rafael F. Coelho

**Affiliations:** 1 Hospital das Clínicas da Faculdade de Medicina da Universidade de São Paulo, SP, Brasil;; 2 Instituto do Câncer do Estado de São Paulo (ICESP), SP, Brasil;; 3 Global Robotics Institute, Orlando, Florida, EUA

## Abstract

**Introduction and objective:**

Retroperitoneal lymph node dissection (RPLND) is indicated for patients with non-seminomatous germ cell tumor (NSGCT) with residual disease after chemotherapy. Although the gold standard approach is still the open surgery, few cases of robot-assisted laparoscopic RPLND have been described. Herein, we aim to present the surgical technique for robot-assisted laparoscopic RPLND.

**Patient and method:**

A 30 year-old asymptomatic man presented with left testicular swelling for 2 months. Physical examination revealed an enlarged and hard left testis. Alpha-fetoprotein (>1000ng/mL) and beta-HCG (>24.000U/L) were increased. Beta-HCG increased to >112.000U/L in less than one month. The patient underwent a left orchiectomy. Pathological examination showed a mixed NSGCT (50% embryonal carcinoma; 30% teratoma; 10% yolk sac; 10% choriocarcinoma). Computed tomography scan revealed a large tumor mass close to the left renal hilum (10x4x4cm) and others enlarged paracaval and paraortic lymph nodes (T2N3M1S3-stage III). Patient was submitted to 4 cycles of BEP with satisfactory response. Residual mass was suggestive of teratoma. Based on these findings, he was submitted to a robot-assisted RPLND.

**Results:**

RPLND was uneventfully performed. Operative time was 3.5 hours. Blood loss was minimal, and there were no intra- or postoperative complications. The patient was discharged from hospital in the 1^st^ postoperative day. Pathological examination showed a pure teratoma. After 6 months of follow-up, patient is asymptomatic with an alpha-fetoprotein of 2.9ng/mL and an undetectable beta-HCG.

**Conclusion:**

Robot-assisted laparoscopic RPLND is a feasible procedure with acceptable morbidity even for post chemotherapy patients when performed by an experienced surgeon.

Available at: http://www.intbrazjurol.com.br/video-section/torricelli_171_171/

